# Diagnostic value of CBCT in Chinese children with adenoid hypertrophy

**DOI:** 10.1002/lio2.837

**Published:** 2022-08-06

**Authors:** Dekun Gao, Xiayu Sun, Ying Yang, Jun Yang, Lan Cheng

**Affiliations:** ^1^ Department of Otorhinolaryngology‐Head and Neck Surgery, Xinhua Hospital Shanghai Jiaotong University School of Medicine Shanghai China; ^2^ Shanghai Jiaotong University School of Medicine Ear Institute Shanghai China; ^3^ Shanghai Key Laboratory of Translational Medicine on Ear and Nose diseases Shanghai China

**Keywords:** adenoid hypertrophy, CBCT, children, NE

## Abstract

**Objective:**

The main objectives of the study were to investigate the reliability and accuracy of cone‐beam computed tomography (CBCT) in the diagnosis of adenoid hypertrophy in Chinese children and to evaluate its value in clinical diagnosis.

**Methods:**

From January 2019 to January 2020, 300 children with sleep snoring in Xinhua Hospital Affiliated to Medical College of Shanghai Jiaotong University were retrospectively studied. All patients underwent nasopharyngoscopy (NE) and CBCT scanning. The sensitivity, specificity, positive predictive value, negative predictive value, positive likelihood ratio, and negative likelihood ratio of CBCT were determined according to the diagnostic criteria of NE, and the consistency between CBCT and NE was evaluated.

**Results:**

The clinical study of 300 children patients found that compared with NE, CBCT had a sensitivity of 87.3%, specificity of 89.2%, the positive predictive value of 93.20%, the negative predictive value of 80.5%, the positive likelihood ratio of 8.08, the negative likelihood ratio of 0.14, and Kappa value of .748.

**Conclusion:**

CBCT is a reliable and accurate tool for the diagnosis of adenoid hypertrophy and can be used as an alternative examination method for children with contraindications or intolerance during NE.

**Level of Evidence:**

4.

## Graphical abstract

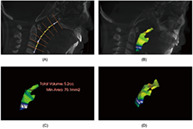

## 1 INTRODUCTION

Adenoids, also known as pharyngeal tonsils, are lymphatic tissues in the nasopharynx that gradually increase in size after birth, reaching a peak at the age of 6–7 years, and gradually shrink with age after 10 years until they disappear. Studies have found that one in 10 healthy children are habitual snorers and one in four snoring children have obstructive sleep apnea syndrome (OSAS),^1^ while adenoid hypertrophy is a very common disorder in pediatric otolaryngology and one of the most common causes of OSAS in children.^2^ An international systematic evaluation and meta‐analysis study found that the prevalence of adenoid hypertrophy in children ranged from 42% to 70%.^3^ Worse still, with the development of society and the increase in obesity and neck circumference in children, the prevalence of adenoid hypertrophy is increasing year by year.^4^

Adenoid hypertrophy leads to reduced ventilation of the pediatric airway with open‐mouth breathing. Long‐term studies have found that reduced ventilation has adverse effects on all systems of the body, as evidenced by neurophysiologic and cognitive deficits^5^ and insidious cardiovascular changes, such as increased pulmonary artery pressure, decreased peak A to peak E flow rate ratio, and increased right ventricular end‐diastolic internal diameter.^6^, ^7^, ^8^ In addition, adenoid hypertrophy affects the hypothalamus to regulate sympathetic and hormonal release, resulting in abnormal secretion of various hormones, such as growth hormone (GH), insulin‐like growth factor‐1 (IGF‐1), Ghrelin, Leptin, and other hormones.^9^ At the same time, the serum Inflammatory factors,^10^ cytokines,^11^ and trace elements^12^ of adenoid hypertrophy are also greatly altered. In addition to these, other effects have not yet been identified and are under investigation.

NE, the traditional diagnostic tool for adenoid hypertrophy, allows visualization of the size of the adenoids and the extent of their obstruction of the posterior nostril through the narrow nasal cavity and structures in the nasal tract and is well used in clinical practice. Currently, NE is the gold standard for the diagnosis of adenoid hypertrophy.^13^ Despite the high accuracy of NE, the young age of the pediatric population and the fear of NE limit its use in the diagnosis of adenoid hypertrophy. Considering these limitations of NE, there are alternative clinical diagnostic methods, one of which is cone‐beam computed tomography (CBCT). CBCT is a 3‐dimensional X‐ray‐based volume‐acquisition imaging method first invented in 1998.^14^ The main advantages of CBCT over conventional CT scans are low radiation dose (40‐fold lower), low cost, fewer artifacts, and the scan time is short and provides continuous thin sections in the coronal, sagittal, and transverse planes. CBCT has been widely used in the diagnosis and treatment of dental diseases since its development.^15^, ^16^ In recent years, CBCT has been gradually used in the diagnosis and treatment of otorhinolaryngological diseases and has a wide range of applications in the detection of mucosal thickness,^17^ nasal septal deviation,^18^ adenoid hypertrophy,^19^ and sinus cysts.^20^ Despite these advances in the application of CBCT in the head and neck region, there is a lack of domestic studies on the accuracy of CBCT diagnosis. The main objective of this study was to investigate the diagnostic concordance between CBCT and NE in pediatric adenoid hypertrophy and the accuracy of CBCT in the diagnosis of patients with adenoid hypertrophy, as well as its feasibility as a noninvasive and painless examination alternative in the examination of adenoids in young children.

## 2 METHODS

### 2.1 Clinical data

This study was conducted from January 2019 to January 2020 at the Department of Otolaryngology‐Head and Neck Surgery, Xinhua Hospital, Shanghai Jiaotong University School of Medicine. The study protocol was approved by the Ethics Committee of Xinhua Hospital, Shanghai Jiaotong University School of Medicine, and all children's parents signed informed consent before enrollment.

The inclusion criteria were:

1. Persistent snoring or mouth breathing for more than 3 months;
2. Children aged between 2 and 10 years old;
3. Have not received any drug or surgical treatment.

The exclusion criteria are:

1. Confirmed diagnosis of various syndromes or the presence of neurological disorders.
2. Previous medication and surgical treatment.
3. Children with craniofacial developmental problems;
4. Patients with excessive hypertrophy of the inferior turbinate that precludes NE.

Finally, we performed NE and CBCT in 300 children aged 2–10 years who met the criteria.

### 2.2 Examination methods

#### 2.2.1 NE

Bilateral nasal cavities were surface anesthetized with a cotton pad containing 1% dicaine without nasal decongestant. 15 min later, a 2.7‐mm diameter Storze 0° scope was used to examine the nasal cavity while the child was awake to observe the extent of adenoidal blockage of the posterior nostril.

#### 2.2.2 CBCT scanning

**FIGURE 1:**
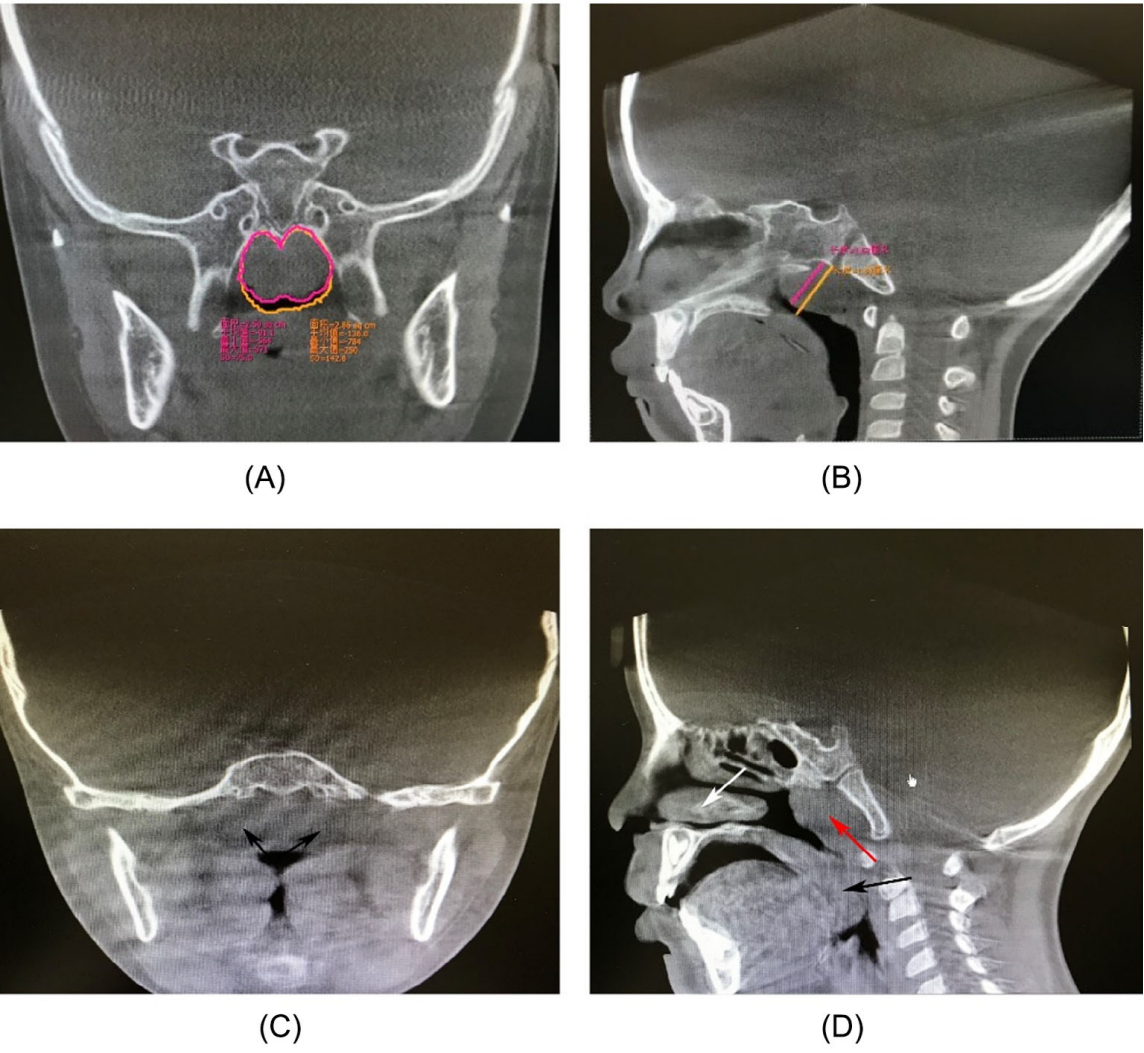
Coronal scan and sagittal reconstruction of CBCT. (A) Coronal scan with hypertrophic adenoids seen at the posterior nostril, the software can automatically calculate the cross‐sectional area (adenoids in the red circle, posterior nostril in the yellow circle). (B) Sagittal reconstruction calculating A/N (red is the thickness of adenoids, yellow is the width of the nasopharyngeal cavity at the most convex part of the adenoids) values. (C) Coronal scan showing hypertrophic tonsils in the oropharynx (black arrows). (D) Sagittal reconstruction showing hypertrophic inferior turbinates (white arrows), hypertrophied adenoids (red arrows), hypertrophied tonsils (black arrows), and airway compression and obstruction.

CBCT was performed immediately after the NE, which is obtained by cone‐beam imaging for 360° scanning. We used a Scanora3Dx CBCT scanner (SBR3D‐2) from Finland. The child was placed in a sitting position while awake, with the head fixed in a brace utilizing a harness, and the CBCT equipment was operated by Scanora software. Technical parameters: cross‐sectional continuous scanning, positioning of the scan marker: frontal centerline is positioned in the mid‐sagittal plane of the body, ranging from the frontal sinus up to the lower edge of the earlobe, and anteriorly to the tip of the nose. Parameters: tube voltage 90 kV, tube current 7 mA, scan time 16.4 s, a field of view (FOV) 140 mm × 165 mm, layer spacing, and layer thickness 0.2 mm. Scan data were reconstructed in coronal and sagittal planes by Invivo software (Figure 1). The radiation dose of CBCT varies depending on the parameters and is generally in the range of 3.9–171.1 μSv, which is equivalent to 2.9 panoramic radiographs (20–100 μSv) on average. CBCT scans can acquire axial, sagittal, and coronal data at the same time. CBCT requires a minimum exposure time of only 2.4 s to obtain an image at standard resolution, which is very fast and is also equipped with a seated fixation system, so the image is essentially free of motion artifacts and breathing rhythm.

#### 2.2.3 Image processing methods of CBCT

**FIGURE 2:**
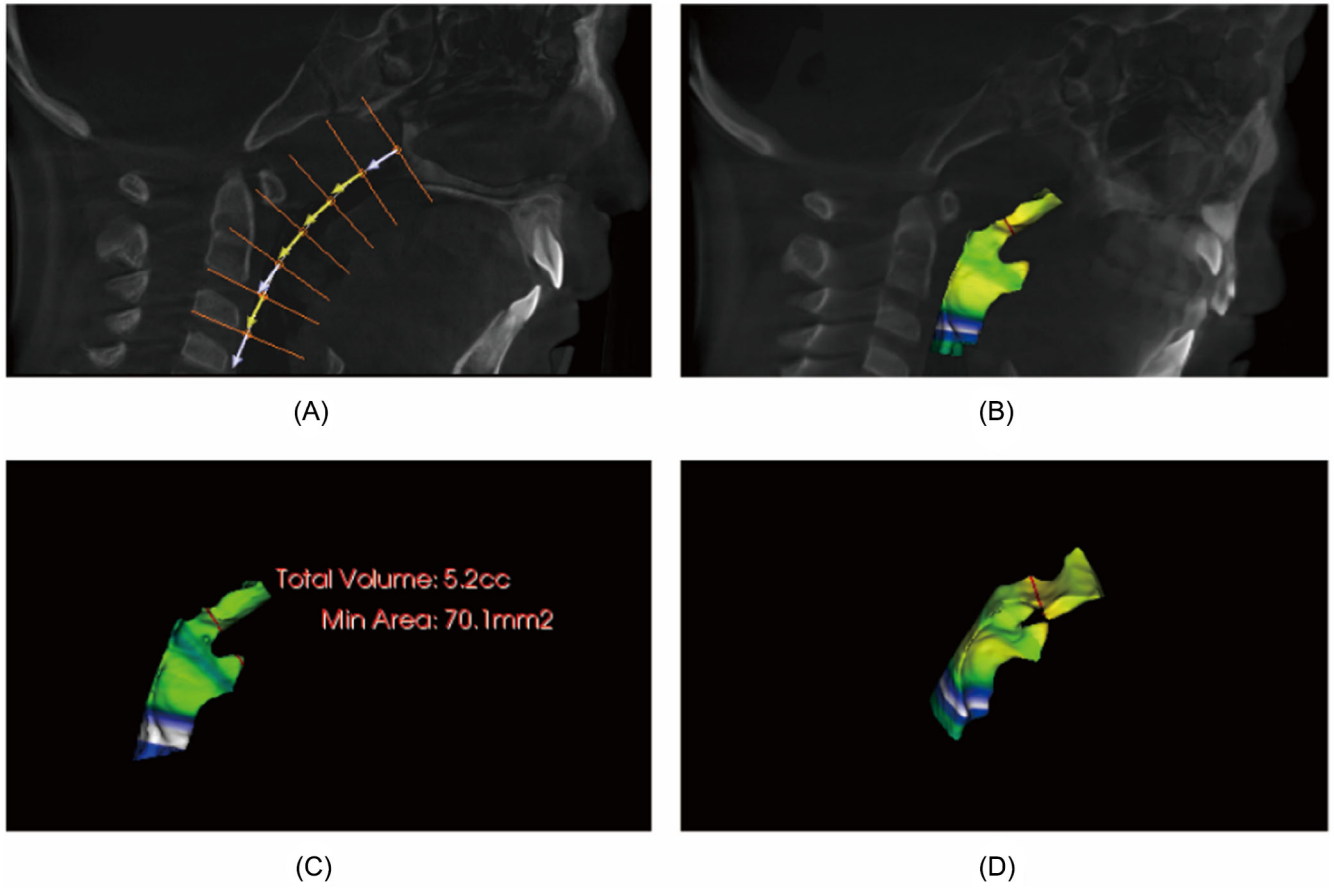
3D airway reconstruction by CBCT. (A) The area between the parietal wall of the nasopharynx and the inferior border of the 3rd cervical vertebra was selected for airway localization. (B) 3D airway angioplasty was performed at the end of localization. (C) Bone and soft tissue artifacts were removed to show the total airway volume and the minimum cross‐sectional area of the posterior nasal aperture with airway compression. (D) The airway was rotated at a certain angle after 3D imaging to show the obvious airway compression and the minimum cross‐section (marked in red).

CBCT can quantitatively determine the degree of adenoid hypertrophy in children. With a coronal scan, the adenoid shadow shown at the posterior nostril is visible, and the degree of its occupation of the posterior nostril can be quickly determined by calculating the cross‐sectional area with the self‐contained software (Figure 1A). Sagittal reconstruction with the same lateral nasopharyngeal slice allows calculation of the A/N ratio (Figure 1B). And the hypertrophied tonsils (1c) can be shown simultaneously. The sagittal reconstruction shows the hypertrophied inferior turbinates (white arrows), hypertrophied adenoids (red arrows), and hypertrophied tonsils (black arrows) on the way from the nasal cavity into the lower airway, showing the airway is blocked by pressure. Post‐processing with Invivo software and 3D reconstruction of the airway allows for a three‐dimensional assessment of airway patency (Figure 2).

#### 2.2.4 Diagnostic criteria

The size of adenoids is measured clinically by observing the degree of adenoidal occlusion of the posterior nostril. In this study, nasal endoscopic observation of ≥75% of the posterior nostril with adenoidal occlusion was considered positive, and CBCT coronal and sagittal reconstructions showed ≥75% of the posterior nostril with adenoidal occlusion were considered positive.

### 2.3 Statistical analysis

Data were expressed as mean ± standard deviation of numerical data, and category data were expressed as number and percentage. Data analysis was performed using SPSS 26.00. Kappa values were used to assess the agreement between the two examination methods, and the significance level was set at *p* < .05.

## 3 RESULTS

The age‐sex distribution of the 300 children is shown in Table 1, and the number of patients in each age group was not statistically different (χ^2^ = 6.478, *p* = .594). The operator of NE and the CBCT imaging reader was the same physicians in all children. CBCT scan revealed 177 children with ≥75% posterior nostril obstruction by adenoids. NE revealed 189 children with ≥75% posterior nostril obstruction by adenoids (see Table 2).

**TABLE 1:** Table of the age and sex of the children

Age (years)	Number		Total
	Male	Female	
2	2	1	3
3	21	33	54
4	30	33	63
5	24	27	51
6	27	15	42
7	21	18	39
8	24	6	30
9	3	3	6
10	6	6	12
Total	159	141	300

**TABLE 2:** Diagnostic results of NE and CBCT for adenoid hypertrophy

	NE		Total
CBCT	Negative	Positive	
Negative	99	24	123
Positive	12	165	177
Total	111	189	300

The sensitivity, specificity, positive predictive value, negative predictive value, positive likelihood ratio, negative likelihood ratio, and Kappa value of CBCT were calculated relative to NE. Using NE as the standard for pediatric adenoid hypertrophy, the sensitivity of CBCT was 87.3% with a 95% confidence interval (CI) of 76.0%–94.0%; the specificity was 89.2% with a 95% CI of 73.6%–96.5%; the positive predictive value was 93.2% with a 95% CI of 82.7%–97.8%; negative predictive value was 80.5%, 95% CI 64.6%–90.6%; positive likelihood ratio was 8.08, 95% CI 3.19–20.47, and negative likelihood ratio was 0.14, 95% CI 0.07–0.27 (see Table 3). In addition, the Kappa values for CBCT and NE compared were 0.748 (*p* < .001), respectively, indicating a good agreement between the two examinations.

**TABLE 3:** Statistical parameters of CBCT compared with NE

Name	Value	95% CI	
Sensitivity	87.30%	76.00%	94.00%
Specificity	89.20%	73.60%	96.50%
Positive predictive value	93.20%	82.70%	97.80%
Negative predictive value	80.50%	64.60%	90.60%
Positive likelihood ratio	8.08	3.19	20.47
Negative likelihood ratio	0.14	0.07	0.27

## 4 DISCUSSION

Symptoms of sleep breathing disorders in children are prevalent, and in the process of clinical diagnosis, we need to promptly identify possible airway obstruction and relieve it. Adenoid hypertrophy has been a common cause of sleep breathing disorders in children for many years, and examination for adenoid hypertrophy has been a hot topic of research because of its unique location that cannot be directly observed. Clinical methods for assessing adenoid hypertrophy have also been developed over many years.^21^ The most traditional method is the lateral nasopharyngeal film, which was gradually replaced by NE with the popularity of endoscopy. In recent years, with the development of imaging technology, various new imaging tests have been used to detect adenoids. CBCT is the most recent technology used for adenoid examination, and several foreign institutions have reported its application in the diagnosis of adenoid hypertrophy disease: Michael P Major examined adenoids using CBCT and NE in children aged 11.5 ± 2.8 years and found good agreement between the two examinations.^22^ Camila Pacheco‐Pereira found that Dolphin Imaging software (CBCT processing software) can be reliable, but it has limited sensitivity and specificity in assessing upper airway obstruction related to adenoid hypertrophy.^23^ CBCT accuracy was moderate to strong when adenoid hypertrophy was grading on a four‐point scale and improved when adenoid hypertrophy was grading on a two‐point scale.^24^ In addition, He found that different levels of CBCT expertise affected the accuracy of the assessment, with a poor agreement between orthodontists' CBCT assessments compared with NE.^19^

Given the above findings, we developed a study in which one physician performed NE and another imaging physician performed CBCT and divided the results of both examinations into a two‐point scale. We found that CBCT has good sensitivity and specificity when compared with NE.

**FIGURE 3:**
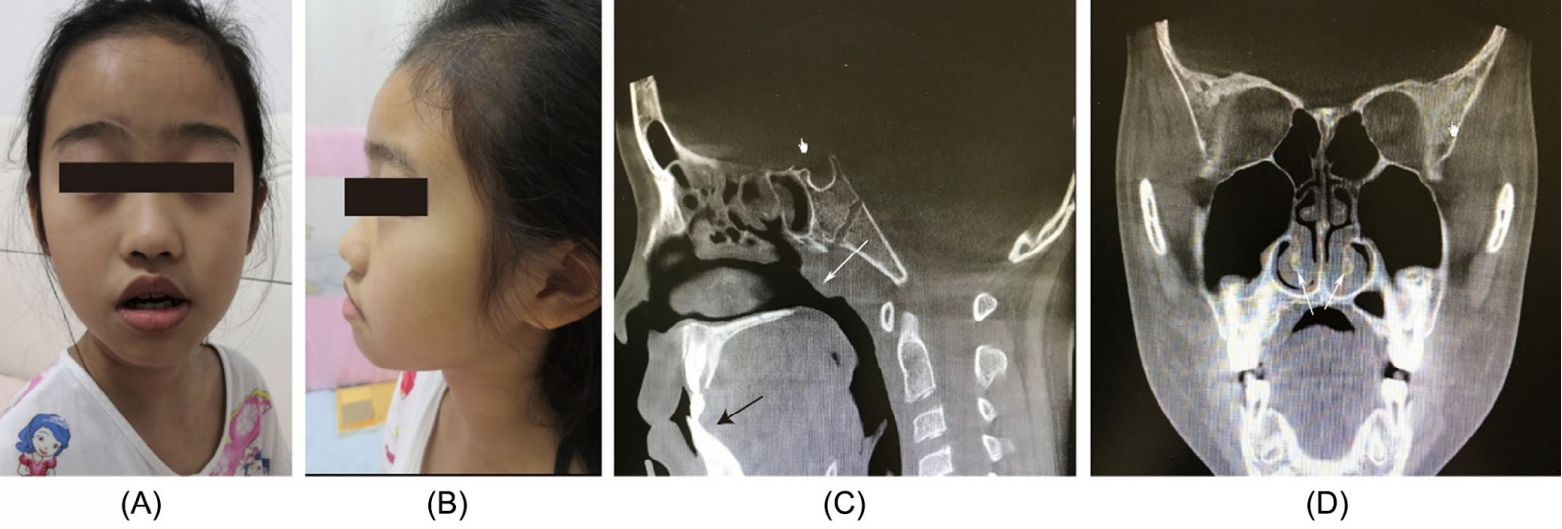
Data of the child in case 1. (A) “Adenoid face” of the child; (B) lateral view of the “adenoid face”. (C) CBCT sagittal reconstruction showing adenoid hypertrophy (white arrows) and dental retrusion (black arrows). (D) Coronal scan showing bilateral inferior turbinate hypertrophy (white arrows)

NE is clear and intuitive and can observe the presence of sinusitis while observing the adenoids, but occasionally in clinical practice, different hospitals give different endoscopic findings. At this point, CBCT can help give an objective and correct judgment. Two typical cases are given below:

Case 1 The child, a 9‐year‐old female, visited two hospitals for “open‐mouth breathing during sleep for 3 years”. Hospital A showed adenoid hypertrophy on endoscopy and recommended surgery; Hospital B showed slight hyperplasia of the adenoids, but the airway was still open and the child was considered too old for surgery. In view of the contrasting diagnoses, the parents came to our department, where a CBCT scan showed bilateral inferior turbinate hypertrophy, adenoid hypertrophy, narrowed airway compression, tonsillar hypertrophy, and dental retrognathism, and recommended tonsil and adenoidectomy, followed by postoperative treatment of allergic rhinitis, correction of mouth breathing, and orthodontic treatment of retrognathism (Figure 3).

**FIGURE 4:**
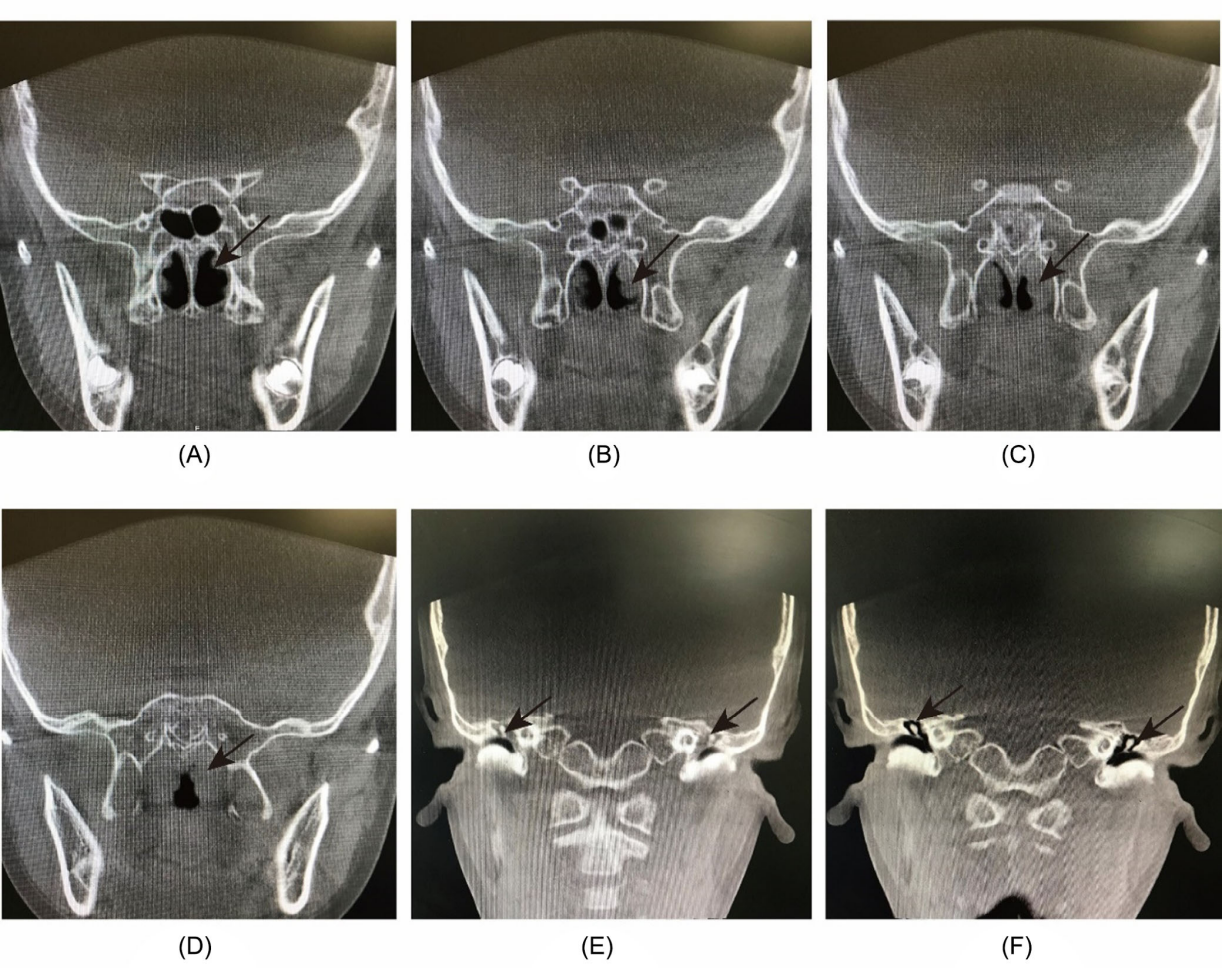
Data of the child in case 2. (A–D) Bilateral abnormal hypertrophy of the nasopharyngeal bullae (black arrows) on serial coronal scans, without adenoid hypertrophy. (E) Bilateral middle ear effusion before treatment (black arrows). (F) Bilateral middle ear effusion absorption after treatment (black arrows)

Case 2 The child, male, 5 years old, had undergone adenoidectomy for “sleep snoring with open mouth breathing” at the age of 3. Two years after the operation, he developed hearing loss in both ears and was diagnosed as “bilateral secretory otitis media”. After 2 months of conservative treatment, the fluid in the middle ear was not absorbed, and NE at the local hospital showed adenoid hypertrophy. His parents thought that the child did not snore or breathe open mouth, so they had doubts about the diagnosis of adenoid recurrence and came to our hospital for a CBCT scan, which showed hyperplasia and hypertrophy of bilateral eustachian tube bullae and bilateral middle ear effusion without significant hypertrophy of adenoids. After giving standardized treatment of eustachian tube dilation and rhinitis, the secretory otitis media was cured after 2 months (Figure 4).

Hyperplasia of the eustachian tube bullae is also common in young children after adenoidectomy. If the child is uncooperative, the endoscope fails to reach the nasopharynx and the abnormally enlarged bullae block the posterior nostril, which can easily be mistaken for hypertrophic adenoids and affect the correct judgment of whether to perform surgery.

There is still a lack of studies related to the diagnosis of adenoid hypertrophy by CBCT in China. Michael P Major published in 2014 about the use of CBCT in adenoid hypertrophy to study the differences between NE and CBCT in the diagnosis of adenoid hypertrophy [25], and the results concluded that CBCT could be used as an alternative tool to NE. However, the age of their study group ranged from 6 to 15 years, while the age stage with a high prevalence of adenoid hypertrophy is 2–10 years, and there is still a lack of reliable data to support this, especially in younger children. In this study, we lowered the age of children to 2 years and focused on the reliability of CBCT compared with NE for the diagnosis of adenoid hypertrophy in a group of children aged 2–10 years. The results showed that CBCT was accurate for assessing adenoid size with good sensitivity, specificity, positive predictive value, and negative predictive value compared with NE, and there was also a strong agreement between the two in examining pediatric adenoid hypertrophy. In addition, CBCT not only visualizes the size of the adenoids but also clearly shows the tonsils blocking the pharyngeal cavity, the sinuses, and the middle ear.

This study concluded that CBCT is highly accurate and reliable in assessing the size of adenoids, but there are some limitations. One of the most critical issues is the radiation from CBCT. Although its radiation is very low, equivalent to 2.9 times of the radiation of X‐ray, it is still harmful to the human body. All parents of the children were informed, and their consent was obtained for this study. We concluded from this study that NE and CBCT are almost identical in the diagnosis of adenoid hypertrophy and can be used clinically as an alternative examination in children who are intolerant to NE. We continue to recommend NE as the preferred screening tool and only inform the children's family of an additional option if it is not available. In addition, our study did not examine the differences between CBCT and other imaging examinations. Despite these drawbacks, our study also revealed the high sensitivity, specificity, and accuracy of CBCT scans and found strong concordance between CBCT and NE in the diagnosis of adenoid hypertrophy.

## 5 CONCLUSION

Considering the low cost, low radiation dose, high sensitivity, and specificity, CBCT may have considerable advantages as a reliable alternative to adenoid examination in children, especially when NE is contraindicated or when younger children are intolerant or uncooperative.

## FUNDING INFORMATION

Shanghai Jiao Tong University Medical‐Industrial Intersection Key Project (No. ZH2018ZDA11).

## CONFLICT OF INTEREST

All authors listed have contributed sufficiently to the project to be included as authors, and all those who are qualified to be authors are listed in the author byline. To the best of our knowledge, no conflict of interest, financial, or other exists.
